# Prevalence of asthma in people with type 1 diabetes mellitus: a scoping review

**DOI:** 10.1186/s13223-024-00869-9

**Published:** 2024-02-08

**Authors:** Júlia Marchatto Kamei, Raissa Dias Maués, Gabriel de Oliveira Silva, Alessandra Helena Machado, Erika Megumi Hoshino, Fabiana Menezes Bacchiega, Laís Mota Furtado Sena, Carlos Antonio Negrato

**Affiliations:** University of São Paulo - Bauru Campus (USP-Bauru), Alameda Dr. Octávio Pinheiro Brisolla, 9-75, Bauru, 17012-901 Brazil

**Keywords:** Prevalence, Asthma, Type 1 diabetes mellitus, Atopy, Autoimmunity, TH1/TH2

## Abstract

**Background:**

According to the Th1/Th2 paradigm, the expansion of Th1-type clones in individuals with type 1 diabetes results in reduced Th2-type clones, preventing the development of atopic diseases and vice versa. However, there is no consensus regarding the direct or inverse relationship between autoimmune and atopic diseases.

**Objective:**

The aim of this scoping review was to examine the knowledge gap about the possibility of coexistence of asthma and type 1 diabetes and determine the prevalence of this association.

**Methods:**

A scoping review was conducted, following the proposal of the Joanna Briggs Institute. The Population, Concept, and Context strategy was used to formulate the guiding question. The proposed question was: “What is the prevalence of asthma in people with T1DM?” After excluding duplicate articles, analyzing titles and abstracts, and excluding articles that did not answer the guiding question, 17 articles remained and were included in this review.

**Results:**

Most of the articles selected conformed to the Th1/Th2 hypothesis, as the prevalence of asthma was lower in individuals with T1DM. However, similar or higher prevalence of asthma was found between cases and controls in few articles.

**Conclusion:**

The prevalence of asthma in people with T1DM ranged from 1.7% to 23.1%. Maybe the mechanisms that characterizes the Th1/Th2 paradigm aren’t as simple as just the interaction of certain cytokines, since Th1-mediated autoimmune diseases and Th2- mediated atopy can coexist.

## Introduction

Asthma and type 1 diabetes mellitus (T1DM) are diseases that have shown an increasing incidence worldwide in recent decades [1, 2]. Both present changes to the immune system, and their appearance depends on an interaction between genetic and environmental factors [3, 4]. Asthma is the most common chronic disease of childhood, while T1DM is one of the most common endocrine diseases found in children and adolescents [1, 2].

Asthma is a heterogeneous disease, usually characterized by chronic airway inflammation [5]. In susceptible individuals, this inflammation causes recurrent episodes of wheezing, breathlessness, chest tightness, and coughing, particularly at night or in the early morning [6]. T1DM is caused by an autoimmune destruction of the pancreatic beta cells, which causes the cessation of insulin production [7, 8]. Regarding the pathophysiology of these diseases, the immune response starts with innate immunity and is followed by adaptive immunity, which can be T Helper 1 (Th1) or T Helper 2 (Th2) type [9]. While Th1 cells secrete interleukins (IL) such as IL-2 and interferon-gamma, Th2 cells secrete IL-4, IL-5, IL-9 and IL-13 [9]. All of these are compensated for by IL-10, that is secreted by regulatory T lymphocytes, which can suppress Th1 and Th2 responses [10].

Genetic factors and an early exposure to antigen help modify the immune response favoring the predominance of Th1 or Th2 responses [9]. While autoimmune diseases with a Th1 response pattern (such as T1DM) are characterized by damage to target organs, those with a Th2 pattern (such as asthma) include allergic and atopic diseases in which high levels of immunoglobulin E (IgE) are found [9]. This is called Th1/Th2 paradigm, according to which the expansion of Th1-type clones in individuals with T1DM would reduce Th2-type clones, preventing the development of atopic diseases and vice versa [11]. According to this concept, diseases characterized by Th1 and Th2 predominance would be mutually exclusive [11].

However, there is no consensus in the literature regarding the existence of a direct or inverse relationship between these two conditions. The aim of this scoping review was to assess the prevalence of asthma in people with T1DM and fill the existing knowledge gap on this topic.

## Methods

### Protocol and eligibility criteria

This is a scoping review that follows the Joanna Briggs Institute criteria for this type of study [12]. This work was structured based on the following steps: (1) preparation of the guiding question and objective of the scoping review; (2) elaboration of the research strategy; (3) literature search in databases; (4) selection of articles based on their titles and abstracts; (5) selection of scientific articles after their full reading; (6) summary of results; and (7) presentation and discussion of these results.

In order to formulate the guiding question for this literature review and research, the Population, Concept and Context (PCC) strategy was used. Therefore, P—population with T1DM; C—patients with T1DM who have asthma, C—in any context. In compliance with the PCC, the following question was elaborated: “What is the prevalence of asthma in people with T1DM?”. The included articles were those that contained the three elements of the PCC strategy, that answered the research question, and that were written in English, Portuguese and Spanish, in any period of time. Articles that were written in other languages, that did not respond to the guiding question, literature reviews and articles whose contents were not found online in full or that belonged to annals of scientific events, were excluded.

### Selection of sources of evidence and data charging process

The search for articles was conducted between September the 2nd and 9th, 2022, with the support of a librarian, in the following databases: PubMed, Scopus, Embase, Web of Science (WoS) and LILACS. Gray literature was not used as a means of searching for articles. Descriptors in health sciences (Decs/Mesh) in Portuguese, English and Spanish were searched, namely, diabetes mellitus tipo 1, type 1 diabetes mellitus, asma, asthma, epidemiologia, epidemiology, prevalência and prevalence. To carry out the search, the Boolean operators OR and AND were used.

**Fig. 1:**
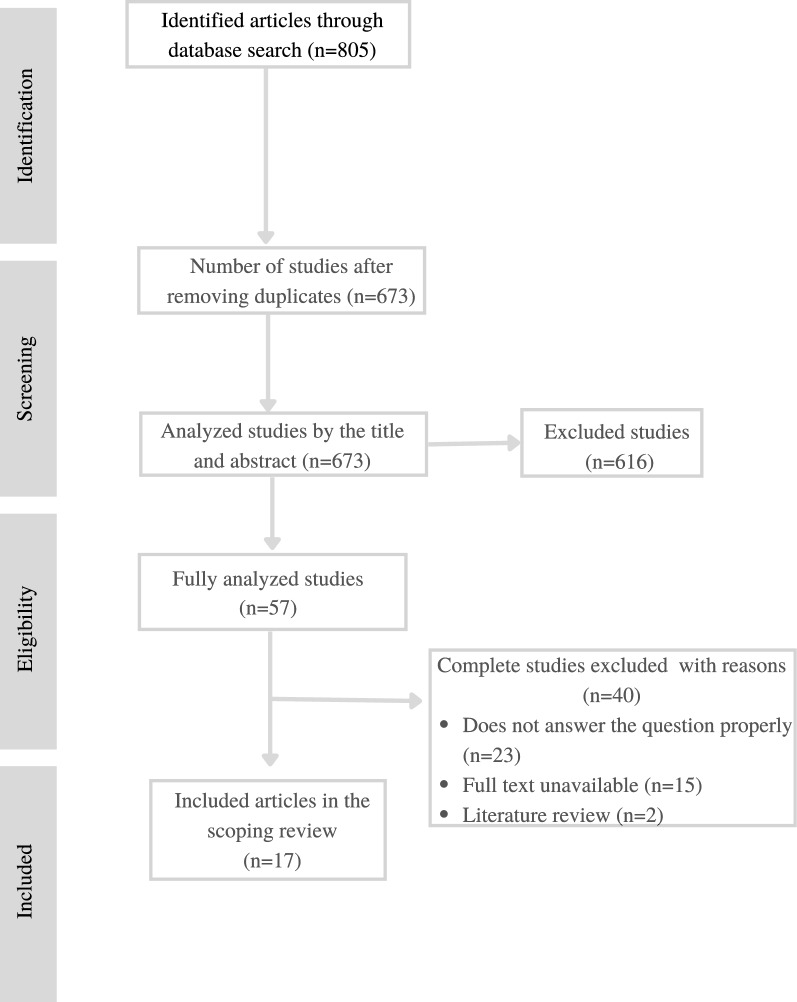
Flowchart of articles identification and selection process

Among the 805 articles found, 132 were excluded with the help of the Mendeley software, since they were duplicated in more than one database. After a careful reading of the titles and abstracts of the 673 remaining articles, 57 were selected for full reading of their contents. After reading these articles in full, 17 were chosen, as they met the pre-established inclusion criteria and constituted the final sample of this work. The selection process was conducted by two independent reviewers (JMK and GOS) and, in case of doubt, a third reviewer (EMH) was consulted. This process is shown in Fig. 1.

For analysis purposes, the articles were numbered from 1 to 17 and named “articles”. The results are presented in the form of tables and reports. To comply with methodological rigor, the Prism tool adapted for the Scoping Review was applied [13].

## Results

### Results related to the main objective

Of the 17 articles included in this review, eight (47.05%) were published in Europe, five (29.41%) in North and South America, three in the Middle East (17.64%) and one in Oceania (5.88%). As for the type of studies, ten (58.82%) were case–control studies, four (23.53%) were cohort studies and three (17.65%) were cross-sectional studies. With regard to the year of publication, five articles (29.41%) were published in 2008, four (23.52%) before and eight (47.05%) after this year. Table 1 presents the articles according to authorship, title, year of publication, journal name, country of publication, type of study, age of evaluated patients and study population.

**Table 1:** Included articles according to authorship, title, year of publication, journal name, country of publication, type of study, age of evaluated patients and study population

Article	Authorship	Title	Year	Journal	Country	Study design	Age group	Population
A1	Petri S Mattila, Jussi Tarkkanen, Harri Saxen et al.	Predisposition to atopic symptoms to inhaled antigens may protect from childhood type 1 diabetes [14]	2002	Diabetes care	USA	Case- control study	T1DM with MA of 18.4 years, siblings without T1DM with MA of 19.2 years, and control with MA of 18.3 years	347 T1DM, 620 unaffected siblings and 483 controls
A2	R Meerwaldt, R J Odink, R Landaeta et al.	A lower prevalence of atopy symptoms in children with type 1 diabetes mellitus [15]	2002	Clinical and experimental allergy: journal of the British Society for Allergy and Clinical Immunology	England	Case- control study	7 to 12 years old	Children with T1DM
A3	Johanna Metsälä, Annamari Lundqvist, Lauri J Virta et al.	The association between asthma and type 1 diabetes: a paediatric case-cohort study in Finland, years 1981–2009 [16]	2018	International Journal of Epidemiology	England	Cohort study	Children up to 16 years old	Children with T1DM or asthma
A4	N. Mostofizadeh, T. Momen, M. Saberi et al.	The Prevalence of Asthma in Children with Type 1 Diabetes Mellitus and Relationship between Control of Diabetes and Severity of Asthma [17]	2018	International Journal of Pediatrics-Mashhad	Iran	Cross-sectional study	Children with MA of 12.65 ± 3.9 years	Children with T1DM
A5	C. Duran, D. Ediger, C. Ersoy et al.	Frequency of atopy and allergic disorders among adults with Type 1 diabetes mellitus in the southern Marmara region of Turkey [10]	2008	Journal of Endocrinological Investigation	Turkey	Case- control study	T1DM with MA of 28.2 ± 8.9 years old and controls with MA of 28.1 ± 5.2 years old	89 T1DM and 64 controls
A6	Vered Gazit, Diana Tasher, Aharon Hanukoglu et al.	Atopy in Children and Adolescents with Insulin-Dependent Diabetes Mellitus [3]	2008	Israel Medical Association Journal	Israel	Case- control study	15 months to 24 years old	65 T1DM and 74 controls
A7	Kyriaki Karavanaki, Eleni Tsoka, Christina Karayianni et al.	Prevalence of allergic symptoms among children with diabetes mellitus type 1 of different socioeconomic status [18]	2008	Pediatric Diabetes	Greece	Case- control study	T1DM children with MA of 10.8 years old (2.46–15.62) and controls with MA of 10.41 years old (1.24–14.50)	127 T1DM and 150 controls
A8	Awad I Smew, Cecilia Lundholm, Lars Sävendahl et al.	Familial Coaggregation of Asthma and Type 1 Diabetes in Children [19]	2020	JAMA Network Open	Sweden	Cohort study	Children	1,284,748 subjects, of whom 121,809 had asthma, 3812 had T1DM, and 494 had asthma and T1DM
A9	Thomas Hörtenhuber, Wieland Kiess, Elke Fröhlich-Reiterer et al.	Asthma in children and adolescents with type 1 diabetes in Germany and Austria: Frequency and metabolic control [20]	2017	Pediatric Diabetes	Austria and Germany	Prospective Cohort study	T1DM under 20 years old	51,926 T1DM, of which 1755 had asthma
A10	Shih-Wen Huang, and Jeff Hitchcock	Influence of the TH1/TH2 Paradigm: The Prevalence of Asthma and Allergic Diseases in Patients with Type 1 Diabetes in the United States [9]	2002	Pediatric Asthma, Allergy & Immunology	USA	Case- control study	T1DM children and unaffected siblings up to 18 years old	403 T1DM, 480 unaffected siblings of T1DM patients and general population as controls
A11	Santhamma James, Angela Pezic, Anne-Louise Ponsonby et al.	Obesity and asthma at school entry: Co-morbidities and temporal trends [21]	2013	Journal of Pediatrics and Child Health	Australia	Cross-sectional study	Children with MA of 5.03 years	7 with asthma and T1DM and 9 control
A12	Mary Helen Black, Andrea Anderson, Ronny A Bell et al.	Prevalence of Asthma and Its Association With Glycemic Control Among Youth With Diabetes [22]	2011	Pediatrics	USA	Cross-sectional study	3 to 21 years old	1683 T1DM and 311 T2DM
A13	Carlo Caffarelli, Giovanni Cavagni, Rossella Pierdomenico et al.	Coexistence of IgE-Mediated Allergy and Type 1 Diabetes in Childhood [23]	2004	International archives of allergy and immunology	Switzerland	Case- control study	T1DM with MA of 12.8 years old (4–19) and controls with MA of 12.2 years old (3–15)	73 T1DM (34 girls and 29 boys) and 108 controls (63 girls and 45 boys)
A14	Murat Cakir, Seker Akcay, Taner Karakas et al.	Prevalence of atopy in children with type 1 diabetes mellitus, hepatitis B virus carriers, and healthy children: role of T helper 1 (Th1)-type immune response [24]	2008	Allergy and Asthma Proceedings	USA	Case- control study	T1DM and HBV children of undefined age and controls aged 10–12 years	52 T1DM (26 boys and 26 girls); 47 HBV carriers (25 boys and 22 girls) and 209 controls (111 boys and 98 girls)
A15	Chris R Cardwell,Dennis J Carson, John Yarnell et al.	Atopy, home environment and the risk of childhood-onset type 1 diabetes: a population-based case–control study [25]	2008	Pediatrics Diabetes	Denmark	Case- control study	Children in two age groups: 6–8 years and 13–14 years	175 T1DM and 4.859 controls
A16	Maria Angela Tosca, Elisa Villa, Michela Silvestri, Giuseppe D'Annunzio et al.	Discrepancy between sensitization to inhaled allergens and respiratory symptoms in pediatric patients with type 1 diabetes mellitus [26]	2009	Pediatric Allergy and Immunology	Italy	Case- control study	T1DM with MA aged 7.8–16.9 years and school-aged controls	112 T1DM (63 boys and 49 girls) and 709 controls
A17	H. Villa-Nova, AM Spinola-Castro, FE Garcia et al.	Prevalence of allergic diseases and/or allergic sensitisation in children and adolescents with type 1 diabetes mellitus [27]	2015	Allergol Immunopathol (Madr)	Brazil	Cohort study	4–18 years old	96 T1DM

HBV, hepatitis B virus; MA, mean age; T1DM, type 1 diabetes mellitus; T2DM, type 2 diabetes mellitus

Our study aimed to fill the knowledge gap regarding the possibility of the coexistence of asthma and T1DM and determine the prevalence of this association; however, our main findings show that there were differences concerning the existence of a direct or inverse relationship between asthma and T1DM. Table 2 shows the relationship found in different studies, as well as the prevalence of asthma in individuals with T1DM, according to age, gender, glycemic control and time since T1DM diagnosis. Seven articles (41.2%) (A1, A2, A3, A4, A10, A13, A14) found an inverse association, that is, a previous diagnosis of T1DM was associated with a reduced prevalence of subsequent asthma, while five articles (29.4%) (A5, A6, A8, A9, A16) found a similar prevalence of asthma among individuals with T1DM and those without diabetes. The direct relationship, which is a higher prevalence of asthma in individuals with T1DM, was observed in only two articles (11.76%) (A11, A17). It is noteworthy, however, that although A8 shows a similar prevalence of asthma among individuals with T1DM and those without diabetes, it shows that people with previous asthma may be at an increased risk of having subsequent T1DM, which is in line with A3. Furthermore, articles A7 and A15 do not specifically address the relationship between T1DM and asthma, but suggest that the prevalence of atopic diseases is similar between individuals with T1DM and those without diabetes.

**Table 2:** Characteristics of the articles based on type of relation between T1DM and asthma, prevalence by age, gender and time diagnosis and considerations about glicemic control

Article	Relationship between asthma and T1DM	Prevalence by age	Prevalence by gender	Glicemic control	Prevalence by time of diagnosis
A1	Inverse relationship	NA	Similar ratio between genders	NA	NA
A2	Inverse relationship	NA	NA	NA	NA
A3	Inverse relationship	T1DM developed more frequently between the ages of 8 to 16 years in children with asthma diagnosed between the ages of 4–7.9 years (0.4%) than in children in good health up to age 8 years (0.3%). Asthma developed less frequently between the ages of 8–16 years in children with T1DM diagnosed between the ages of 4–7.9 years (1.0%) than in those in good health up to age 8 years (1.3%)	Boys had a higher risk of asthma and T1DM compared to girls	T1DM children with asthma have worse glycemic control	NA
A4	Inverse relationship	NA	There is a higher proportion of diabetics with asthma in males (18 males vs. 6 females), which is similar to the predominance of asthmatic males among healthy children	In patients with T1DM, glycemic control was worse in those with asthma than in those without asthma	NA
A5	Similar prevalence	NA	NA	NA	NA
A6	Similar prevalence	NA	NA	NA	NA
A7	NA	NA	NA	NA	NA
A8	Similar prevalence of asthma in T1DM Increased prevalence of T1DM in asthmatics	NA	Asthma and T1DM are in 60.5% of boys and 39% of girls	NA	NA
A9	Similar prevalence	Patients with T1DM and asthma were older than those with only T1DM	Patients with T1DM and asthma were more often male than those with only T1DM	No differences in HbA1c of the groups with T1DM and asthma / only T1DM. However, patients with concomitant asthma need more insulin	NA
A10	Inverse relationship	NA	NA	NA	NA
A11	Direct relationship	NA	NA	NA	NA
A12	NA	Asthma was predominant in older patients	Asthma was more prevalent in males	T1DM patients with asthma have worse glycemic control	NA
A13	Inverse relationship	NA	NA	NA	In 14 diabetic children, the mean age of onset of allergic symptoms was 3.42 ± SD 3.45, similar to that of the controls (5.90 ± SD 3.93; p = 0.083)
A14	Inverse relationship	NA	NA	NA	NA
A15	NA	Higher number of asthma cases in those in older age groups (13–14 years) compared to the control group (13–14 years)	NA	NA	NA
A16	Similar prevalence	NA	NA	Glycemic control was measured according to the patients' HbA1c and was not correlated with asthma severity	NA
A17	Direct relationship	NA	NA	NA	At least four years, the duration of T1DM had no impact on the frequency of allergic disease: 61.7% (29/47) had T1DM for more than four years

HbA1c, hemoglobin A1c; NA, not applicable

### Other results

In addition to the main findings, out of the six articles that addressed prevalence by sex, five of them (83.33%) (A3, A4, A8, A9 and A12) showed that the coexistence of both diseases was more frequent in males. As for glycemic control, two articles (11.76%) (A4, A12) showed that it was worse in patients who had T1DM and asthma concomitantly. However, A9 showed that there was no difference in glycosylated hemoglobin levels (HbA1c) between patients with T1DM and asthma compared to patients with only T1DM. However, patients with asthma and T1DM needed higher doses of insulin for reaching a good glycemic control. A16 showed that glycemic control was not associated with asthma severity. Two articles (11.76%) (A13 and A17) addressed the issue of asthma prevalence in patients with T1DM, taking into account the time of diagnosis of T1DM. A13 showed that most children with T1DM had a history of allergic symptoms before the onset of T1DM, with a time of onset of allergic symptoms similar to that of controls. According to A17, the duration of T1DM had no impact on the frequency of allergic diseases, since 61.7% of the patients (29/47) had T1DM for more than four years and 67.3% had T1DM for less than four years.

Table 3 shows the prevalence of asthma in patients with T1DM and of T1DM in patients with asthma, which was described in sixteen (94.11%) and three articles (17.64%), respectively. Furthermore, among the analyzed articles, two (11.76%) described both prevalences. Among the case–control studies, only two (20%) found a higher prevalence of asthma in individuals with T1DM, and in A15 this situation was found only in the group with the highest age group. Article A10 compared the prevalence of asthma in individuals with T1DM, in the control group and in siblings of individuals with T1DM, which was 4.4%, 9.4% and 7.2%, respectively. Article A14 compared the prevalence of asthma in individuals with T1DM, in a control group and in individuals with hepatitis B, being 9.6%, 12.4% and 31.9%, respectively. Of the cohort studies, A3 and A8 found that the prevalence of asthma in individuals with T1DM and of T1DM in individuals with asthma, was 1.7% and 0.6% in A3 and 13% and 0.30% in A8, respectively. A17 showed a prevalence of 22.90% of asthma in individuals with T1DM. Among the cross-sectional studies, A4 showed a higher prevalence of asthma in the control group than in individuals with T1DM, A11 showed a higher prevalence of T1DM in individuals with asthma, and, finally, study A12 found a 10% prevalence of asthma in a cohort of 1,683 individuals with T1DM.

**Table 3:** Prevalence of asthma in T1DM patients and prevalence of T1DM in asthmatic patients

Article	Title	Prevalence of asthma in T1DM patients (P = Population with T1DM; C = controls)	Prevalence of T1DM in asthmatic patients (P = population with asthma; C = controls)
A1	Predisposition to atopic symptoms to inhaled antigens may protect from childhood type 1 diabetes	P: 4,1% C:6,7%	NA
A2	A lower prevalence of atopy symptoms in children with type 1 diabetes mellitus	P: 17,1% C:22,5%	NA
A3	The association between asthma and type 1 diabetes: a pediatric case-cohort study in Finland, years 1981–2009	P: 1,7%	P: 0,6%
A4	The Prevalence of Asthma in Children with Type 1 Diabetes Mellitus and Relationship between Control of Diabetes and Severity of Asthma	P: 5,7% C:12,3%	NA
A5	Frequency of atopy and allergic disorders among adults with Type 1 diabetes mellitus in the southern Marmara region of Turkey	P: 3,4% C: 3,1%	NA
A6	Atopy in Children and Adolescents with Insulin-Dependent Diabetes Mellitus	P: 23,1% C: 29,7%	NA
A7	Prevalence of allergic symptoms among children with diabetes mellitus type 1 of different socioeconomic status	P: 13,64% C: 15,65%	NA
A8	Familial Coaggregation of Asthma and Type 1 Diabetes in Children	P:13%	P: 0,3%
A9	Asthma in children and adolescents with type 1 diabetes in Germany and Austria: Frequency and metabolic contro	P: 3,38%	NA
A10	Influence of the TH1/TH2 Paradigm: The Prevalence of Asthma and Allergic Diseases in Patients with Type 1 Diabetes in the United States	P: 4,4% C: 9,4%;	NA
A11	Obesity and asthma at school entry: Co-morbidities and temporal trends	NA	P: 0,4% C:0,06%
A12	Prevalence of Asthma and Its Association With Glycemic Control Among Youth With Diabetes	P:10%	NA
A13	Coexistence of IgE-Mediated Allergy and Type 1 Diabetes in Childhood	P: 3.1% C: 14,8	NA
A14	Prevalence of atopy in children with type 1 diabetes mellitus, hepatitis B virus carriers, and healthy children: role of T helper 1 (Th1)-type immune response	P: 9,6% C: 12,4% HBV carriers: 31,9%	NA
A15	Atopy, home environment and the risk of childhood-onset type 1 diabetes: a population-based case–control study	Younger group: P 19%, C 22% Older group: P 19%, C 18%	NA
A16	Discrepancy between sensitization to inhaled allergens and respiratory symptoms in pediatric patients with type 1 diabetes mellitus	P:14,3% C:16,5%	NA
A17	Prevalence of allergic diseases and/or allergic sensitisation in children and adolescents with type 1 diabetes mellitus	P: 22,9%	NA

NA, not applicable

## Discussion

### Data related to the objective

According to the Th1/Th2 paradigm, there would be an inverse relationship between the prevalence of T1DM and asthma [11]. This would occur since Th1 and Th2 cells can inhibit each other by secreting cytokines, so that asthma and allergic diseases are supposed to appear less frequently in patients with Th1 mediated autoimmune diseases [10]. Among the articles that were included in this review, seven conformed with this paradigm, five showed that the prevalence of asthma is similar between patients with and without T1DM, and two found a direct relationship between asthma and T1DM, suggesting that the interaction between Th1 and patterns Th2 is more complex than initially proposed.

Of the articles that showed an inverse relationship between the two conditions, A1 showed that the existence of T1DM was inversely associated with asthma and hypersensitivity to allergens, compared to individuals in the control group. A2 observed a lower prevalence of asthma and no association with the presence of atopic diseases in patients with T1DM compared to controls. A3 found that the relationship between the two diseases depends on their order of appearance, with a previous diagnosis of asthma increasing the risk of T1DM by 41%, while a prior diagnosis of T1DM decreased the risk of asthma by 18%. In the same way, A8 pointed out that children with asthma had an increased risk of T1DM later on, however, the subsequent risk of asthma did not differ substantially between children with T1DM and controls. A4 pointed that the prevalence of asthma in patients with T1DM is approximately half that found in the general population (5.7% vs 12.3%). Some studies show that the prevalence of asthma and rhinitis is lower in individuals with T1DM than in control groups (A10, A13 and A14). These examples of inverse relationship indicate the protective role of Th1 cells for allergic diseases [10] and suggest that, overall, when the dominant immunologic response is enhanced by cytokines of Th1 cells, this diminishes the effect of cytokines from Th2 cells in the same host, so that the end result is that in patients with Th1 driven diseases, Th2 driven diseases are usually not found [9].

However, some studies have shown that the Th1/Th2 paradigm does not seem to actually occur, so that there may be coexistence of cytokines from both patterns in the development of both diseases, with complex interactions that have not yet been fully elucidated. Thus, some analyzed articles showed similar frequencies of asthma in individuals with and without T1DM (A5, A6, A9, A16). Other studies, despite not specifically establishing the relationship between T1DM and asthma, point out that the prevalence of atopy is similar between individuals with and without T1DM (A7, A15). These examples of studies are in contrast with the “traditional” concept of an inverse association between atopy and autoimmunity, and some evidences have shown that autoimmune Th1 diseases such as T1DM, thyroiditis and psoriasis in both adults and children could coexist with Th2 mediated diseases, suggesting that the Th1/Th2 paradigm is oversimplified [3].

There was also an article (A11) that found the presence of a direct relationship between T1DM and asthma, since it was found that children with T1DM are more likely to have asthma, however, A17 reported a similar frequency of sensitization to allergens in children with and without T1DM.

### Data related to epidemiology rather than pathophysiology

With regard to body mass index (BMI), it was found that patients with T1DM and concomitant asthma had a higher BMI than those who had asthma alone (A9, A12). This association can be explained by the existing inflammatory process in obesity that would precipitate the onset of asthma in individuals with T1DM (A12).

Regarding sex, males were the most affected among patients who had both conditions concomitantly or T1DM alone (A3, A4, A8, A9). However, in A17, gender did not influence the development of allergic symptoms or the prevalence of allergic diseases in those individuals with T1DM.

A complex familial relationship was found between asthma and T1DM. A1 showed that the frequency of T1DM in relatives was inversely associated with that of asthma. A8 showed that relatives of individuals with asthma or T1DM have an increased risk of developing both diseases, with this risk being greater among siblings of the same father and mother and more attenuated among cousins and half-siblings. This suggests that there are shared genetic and/or environmental factors that contribute to the development of both diseases.

Articles A4 and A12 showed that individuals with asthma and T1DM had worse glycemic control when compared to patients with only T1DM. The A4 study that was carried out with Iranian children with T1DM classified glycemic control, through the measurement of glycated hemoglobin, as good in 33.9%, moderate in 53.5% and poor in 12.6%. Those individuals who had asthma and T1DM, glycemic control was worse, with the prevalence of good control in 25%, moderate in 50% and poor in 25%, respectively. The same was found in study A12, where among young people with T1DM, asthma was associated with poor glycemic control, especially if glycemic control was inadequate (approximately 31%). However, there does not seem to be unanimity regarding this proposition. Study A9, carried out with children and adolescents from Germany and Austria, did not find differences in glycemic control between groups with T1DM and asthma and only with T1DM. However, the insulin doses used by individuals with concomitant asthma and T1DM were higher.

Study A9 identified that patients with T1DM and asthma had higher occurrence of diabetic ketoacidosis among those who used inhaled sympathomimetics compared to those who used inhaled corticosteroids. In this way, asthma management seems to have an influence on the outcomes of T1DM. Furthermore, it is worth noting that, in this study, no difference was found comparing diabetes-related complications in individuals using all other asthma medications.

Articles A4 and A12 showed a significant correlation between parental education and diabetes control. Studies A4, A9 and A12 verified that the development or not of asthma is not influenced by the educational level of the parents.

### Limitations

Although following an established methodology, both for the search and for the identification of published literature, this scoping review has some limitations that should be mentioned, since some information may have been omitted, as articles that were not written in English, Spanish or Portuguese were not included. Gray literature was also not accessed, and PubMed, Scopus, Embase, Web of Science (WoS) and LILACS were the only databases consulted. There was also a great difference between the studied populations, the number of participants, the age groups and the methodology of the analyzed articles.

## Conclusion

In conclusion, based on the assumption that the expansion of Th1 clones in individuals with T1DM would cause a reduction in the Th2 response, preventing the development of atopic diseases and vice versa [28], most of the articles included in this scoping review converged on this hypothesis (A1, A2, A3, A4, A10, A13, A14). However, some articles diverged from this assumption so that a similar or higher prevalence of asthma was found in cases and controls (similar prevalence: A5, A6, A8, A9, A16 and higher prevalence: A11, A17). The prevalence of asthma in people with T1DM ranged from 1.7% to 23.1%. Based on our findings, the mechanisms that permeate the Th1/Th2 paradigm, in which T or other types of lymphocytes control the type of immune responses generated by the profile of cytokines they secrete, it was observed that there can be an interaction or simultaneous presence of different types of immune responses in the same individual, so that Th1-mediated autoimmune diseases and Th2-mediated atopic diseases can coexist. Future randomized and controlled trials with greater number of participants and longer duration lasting should be carried out to a better comprehension of this topic.

## Data Availability

The datasets generated and/or analysed during the current study are available in articles of PubMed, Scopus, Embase, Web of Science (WoS) and LILACS by using descriptors such diabetes mellitus tipo 1, type 1 diabetes mellitus, asma, asthma, epidemiologia, epidemiology, prevalência and prevalence. To carry out the search, the Boolean operators OR and AND were used. The datasets used and/or analysed during the current study are available from the corresponding author on reasonable request. All data generated or analysed during this study are included in this published article. The datasets, as the tables and methods’s fluxogram generated and/or analysed during the current study are not publicly available due to it is material that was made up by the authors of the review themselves but are available from the corresponding author on reasonable request.

## Abbreviations

MA: Mean age
Y: Years
T1DM: Type 1 diabetes mellitus
T2DM: Type 2 diabetes mellitus
HBV: Hepatitis B virus
NA: Not applicable
HbA1c: Glycated hemoglobin
